# The Brier score does not evaluate the clinical utility of diagnostic tests or prediction models

**DOI:** 10.1186/s41512-017-0020-3

**Published:** 2017-12-02

**Authors:** Melissa Assel, Daniel D. Sjoberg, Andrew J. Vickers

**Affiliations:** Department of Epidemiology and Biostatistics, Memorial Sloan Kettering Cancer Center, New York, USA

**Keywords:** Prediction modeling, Concordance index, Sensitivity, Specificity, Net benefit, Brier score, Mean squared error

## Abstract

**Background:**

A variety of statistics have been proposed as tools to help investigators assess the value of diagnostic tests or prediction models. The Brier score has been recommended on the grounds that it is a proper scoring rule that is affected by both discrimination and calibration. However, the Brier score is prevalence dependent in such a way that the rank ordering of tests or models may inappropriately vary by prevalence.

**Methods:**

We explored four common clinical scenarios: comparison of a highly accurate binary test with a continuous prediction model of moderate predictiveness; comparison of two binary tests where the importance of sensitivity versus specificity is inversely associated with prevalence; comparison of models and tests to default strategies of assuming that all or no patients are positive; and comparison of two models with miscalibration in opposite directions.

**Results:**

In each case, we found that the Brier score gave an inappropriate rank ordering of the tests and models. Conversely, net benefit, a decision-analytic measure, gave results that always favored the preferable test or model.

**Conclusions:**

Brier score does not evaluate clinical value of diagnostic tests or prediction models. We advocate, as an alternative, the use of decision-analytic measures such as net benefit.

**Trial registration:**

Not applicable.

## Background

A variety of statistics have been proposed as tools to help investigators evaluate diagnostic tests and prediction models. Sensitivity and specificity are generally reported for binary tests; for prediction models that give a continuous range of probabilities, discrimination (area under the curve (AUC) or concordance index) and calibration are recommended [1].

Recent years have seen considerable methodologic criticism of these traditional metrics. This was driven, at least in part, by interest in molecular markers. For instance, it has been argued out that AUC is insensitive, that it does not markedly increase when a new marker is added to a model unless the odds ratio for that marker is very high [2, 3]. In 2008, Pencina and colleagues introduced the net reclassification improvement (NRI) as an alternative metric to AUC [4]. The NRI measures the incremental prognostic impact of a new predictor when added to an existing prediction model for a binary outcome [5]. The metric became very widely used within a short period of time, a phenomenon attributed to the view that change in AUC will be small even for valuable markers [6]. The NRI has since been debunked; Kerr et al. provide a comprehensive evaluation of the disadvantages of NRI [7]. Hilden and Gerds demonstrated that miscalibration can improve NRI and so, critically, this results in the size of the test for NRI being much larger than nominal levels under the null [5]. Hilden and Gerds’ commentary on their findings focuses on the concept of a “proper scoring rule”, that is, a metric that is maximized when correct probabilities are used [5]. The authors mention the Brier score as an example of a proper scoring rule.

The Brier score is an improvement over other statistical performance measures, such as AUC, because it is influenced by both discrimination and calibration simultaneously, with smaller values indicating superior model performance. The Brier score also estimates a well-defined parameter in the population, the mean squared distance between the observed and expected outcomes. The square root of the Brier score is thus the expected distance between the observed and predicted value on the probability scale.

Hence, the Brier score would appear to be an attractive replacement for NRI, and it has indeed been recommended and used in statistical practice to evaluate the clinical value of tests and models. For instance, the Encyclopedia of Medical Decision Making describes the use of the Brier score in the “Evaluation of statistical prediction rules” [Binder H, Graf E. Brier scores. In: [8]]. As a practical example, a study by la Cour Freiesleben et al. aimed to develop prognostic models for identification of patients’ risks of low and excessive response to conventional stimulation for in vitro fertilization/intracytoplasmic sperm injection in order to ascertain if a low or a high dosage level should be used. The conclusions concerned “the best prognostic model” for each of the two endpoints, with models that were selected on the basis of Brier score. The authors then recommend that the models “be used for evidence-based risk assessment before ovarian stimulation and may assist clinicians in individual dosage [between two alternatives] of their patients” [9]. This is a clear example where authors used the Brier score to make clinical recommendations.

The Brier score has also been used to evaluate binary diagnostic tests. For instance, Braga et al. [10] compared six binary decision rules for Zika infection with a novel prediction model that provided a semi-continuous score. Brier scores were reported for all comparators. The authors stated that the “lowest Brier score of 0.096” was for the prediction model, leading to the conclusion that “the model is useful for countries experiencing triple arboviral epidemics”. Similarly, Kloeckner et al. [11] used Brier scores to compare two binary risk groupings with a three-group categorization for survival after chemoembolization for liver cancer. They concluded that risk groupings were not “sufficient to support clear-cut clinical decisions”.

The Brier score depends on prevalence in such a way [12] that it may give undesirable results where clinical consequences are discordant with prevalence. For instance, if a disease was rare (low prevalence), but very serious and easily cured by an innocuous treatment (strong benefit to detection), the Brier score may inappropriately favor a specific test compared to one of greater sensitivity. Indeed, this is approximately what was seen in the Zika virus paper [10], where the test with high sensitivity and moderate specificity (81 and 58%) had a much poorer Brier score than a test with low sensitivity but near perfect specificity (29 and 97%).

In this paper, we investigate scenarios in which we anticipate the Brier score might give a counter-intuitive rank ordering of tests and models. If the Brier score performs poorly in at least some common scenarios, this refutes any claim that it has general value as a metric for the clinical value of diagnostic tests or prediction models. As a comparator, we apply a decision-analytic net benefit method to the same scenarios. We start by introducing the Brier score and the decision-analytic alternative before applying both to four illustrative scenarios.

### Brier score

The Brier score was introduced by Brier in 1950 to address the issue of verification of weather forecasts and has since been adopted outside the field of meteorology as a simple scoring rule for assessing predictions of binary outcomes. The Brier score was a measure developed to scale the accuracy of weather forecasts based on Euclidean distance between the actual outcome and the predicted probability assigned to the outcome for each observation [13]. The Brier score simultaneously captures discrimination and calibration, with low values being desirable.

It has been previously established that the Brier score is a proper scoring rule [14]. As overfitting results in miscalibration, this property penalizes overfit. For instance, Hilden and Gerds generated regression trees (“greedy” and “modest”) from a training dataset with varying propensities for overfit. When the models were applied to a validation set, the Brier score was superior for the modest tree, although the NRI favored the greedy tree [5].

In terms of notation, *D* is a random variable representing the outcome and *X* is a random variable representing the predicted probability of the outcome. Consider a set of *n* patients, let the subscript *i* index the individual patient. Let *d*_*i*_ represent the observed outcome of patient *i*, such that *d*_*i*_ = 0 if the disease is absent and *d*_*i*_ = 1 if the disease is present. Let *x*_*i*_ denote the predicted probability of the disease corresponding to the *i*th patient. The Brier score, the mean squared prediction error, is defined as:

$$ \mathrm{BS}\left(D,X\right)=E{\left[D-X\right]}^2 $$

The expected value of the Brier score can be estimated by using $\frac{1}{n}{\sum}_{i=1}^n{\left({d}_i-{x}_i\right)}^2$ provided that 1 ≥ *x*_*i*_ ≥ 0 for all *i* = 0, 1, 2,…, *n*.

We wish to calculate Brier scores for several hypothetical scenarios where we vary the prevalence and calibration of a model. In the case of a binary test, let *T*_*i*_ denote the result of the test corresponding to the *i*th patient, such that *T*_*i*_ = 1 if the test is positive for the disease and *T*_*i*_ = 0 if the test is negative. The expected Brier score can be represented by:

$$ E\left[\mathrm{BS}\right]=P\left(T=1,D=0\right)+P\left(T=0,D=1\right) $$

This equals the misclassification rate in this binary test setting. We will refer to this derivation as “method 1”. An alternative to viewing a binary test as giving probabilities of 0 or 1 is to use the probability of disease among the test positive (positive predictive value) for a positive test and the probability of disease among the test negative cases (one minus the negative predictive value) for a negative test. We will refer to this derivation as “method 2”. This gives an expected Brier score:

$$ E\left[\mathrm{BS}\right]={\left(1-\mathrm{PPV}\right)}^2P\left(D=1,T=1\right)+{\mathrm{PPV}}^2P\left(D=0,T=1\right)+{\mathrm{NPV}}^2P\left(D=1,T=0\right)+{\left(1-\mathrm{NPV}\right)}^2P\left(D=0,T=0\right) $$

Method 1 might therefore be seen as a miscalibrated version of method 2. In the case of logistic regression, the Brier score can be written as a function of *z*, a continuous covariate, and the slope coefficients.

$$ \log \left(\frac{P\left(D=1|Z=z\right)}{1-P\left(D=1|Z=z\right)}\right)=\mathrm{logit}\left)P\left(D=1|Z=z\right)\right)={\beta}_0+{\beta}_1z,\mathrm{where}Z\sim {f}_{\mathrm{Z}}\left(\mathrm{z}\right) $$

$$ D\mid Z=z\sim \mathrm{Bernoulli}\left(\mathrm{logi}{\mathrm{t}}^{-1}\left({\beta}_0+{\beta}_1z\right)\right) $$

The Brier score can be represented using the joint distribution of *D* and *X*, where *X* = logit^−1^(*β*_0_ + *β*_1_ *z*).

$$ \mathrm{BS}\left(D,X\right)=E{\left[D-X\right]}^2=\sum \limits_{d=0}^1\underset{x=0}{\overset{1}{\int }}{\left(d-x\right)}^2{f}_{DX}\left(d,x\right) dx $$

where

$$ {f}_{DX}\left(d,x\right)={f}_{DZ}\left(d,z\right)\frac{{\left|{\beta}_1\right|}^{-1}}{x\left(1-x\right)}\kern0.5em ,\mathrm{and}\kern0.35em {f}_{DZ}\left(d,z\right)={f}_{D\mid Z=z}(d){f}_Z(z) $$

Therefore, the value of the Brier score in the case of logistic regression can be directly calculated using the following equation:

$$ \mathrm{BS}\left(D,X\right)=\sum \limits_{d=0}^1{\int}_{x=0}^1{\left(d-x\right)}^2\frac{{\left|{\beta}_1\right|}^{-1}}{x\left(1-x\right)}{x}^d{\left(1-x\right)}^{1-d}{f}_Z(z) dx,\mathrm{where}\ x=\frac{1}{1+{e}^{-\left({\beta}_0+{\beta}_1z\right)}} $$

### Net benefit

Net benefit is a decision-analytic statistic that incorporates benefits (true positives) and harms (false positives), weighting the latter to reflect relative clinical consequences [15]. Net benefit is often reported as a decision curve, where net benefit is plotted against threshold probability, *p*_t_ defined as the minimum probability of disease $\widehat{p}$ at which a patient will opt for treatment [16]. For example, a 5% threshold probability means that if a patient’s risk of disease is 5% or more, the patient should be treated; if it is less than 5%, treatment should be avoided. In other words, a threshold probability of 5% means that if a disease went untreated, it would be 19 times worse than an unnecessary treatment. Net benefit has been shown to be a proper scoring rule, as any difference between the true probability of the event and the predicted probability decreases net benefit [17, 18].

We wished to compare net benefit at various threshold probabilities of interest for various binary tests and continuous prediction models in our example scenarios [16]. The net benefit of the risk prediction weighs the relative harm of a false-positive and a false-negative result based on a desired threshold probability [16].

$$ \mathrm{Net}\ \mathrm{benefit}=\mathrm{TPR}-\mathrm{FPR}\left(\frac{p_{\mathrm{t}}}{1-{p}_{\mathrm{t}}}\right) $$

## Methods

### Illustrative scenarios

Consider we wish to examine the performance of the Brier score based on a logistic regression model. In our primary example, we used an event rate of 20% and a prediction model with an AUC close to 0.75 that outputs probabilities that are normally distributed on the logit scale.

$$ \mathrm{logit}\left(P\left(D=1|Z=z\right)\right)=-1.65+z,\mathrm{where}Z\sim N\left(0,1\right) $$

This model was then intentionally miscalibrated by shifts on the intercept coefficient (*γ*) to assess how miscalibration affects the Brier score and the net benefit.

$$ \mathrm{logit}\left(P\left(D=1|Z=z\right)\right)=-1.65+\gamma +z $$

**Fig. 1:**
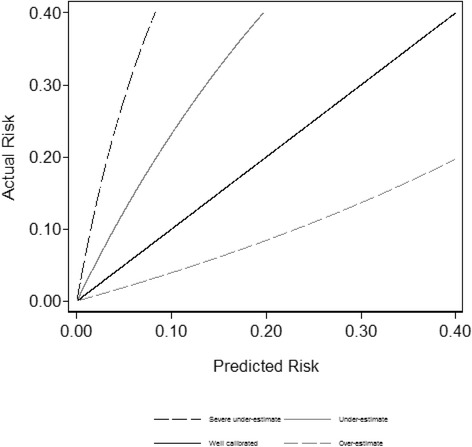
Calibration plot for various continuous prediction models of differing degrees of miscalibration. All prediction models have an AUC of 0.75 for predicting an event with prevalence 20%. The prediction models include the following: a well-calibrated prediction model, a model that is miscalibrated such that it overestimates risk, a prediction model that underestimates risk, and a prediction model that more severely underestimates risk

First, *γ* was varied from − 2, − 1, 0, to 1 yielding predictions that severely under predicted, under predicted, well calibrated, and over predicted the true probability of the outcome, respectively. The calibration plots corresponding to each of these scenarios are shown in Fig. 1.

We also assumed the availability of two binary tests, one with sensitivity 95% and specificity 50% and another with sensitivity 50% and specificity 95%. In the case of the binary tests, the AUC is assessed by first constructing the non-parametric ROC curve. For binary data, the sensitivity and specificity are calculated at 3 points: ≥ 0, ≥ 1, and > 1. The first is where sensitivity is 100%, and the last is where specificity is 100%. The 3 points are plotted, connected by straight lines, and the AUC is calculated using the trapezoidal rule.

## Results

### Scenarios demonstrating counterintuitive results from Brier score

#### Comparison of two binary tests

Assume a clinical scenario where the event is fatal unless identified early but can be easily cured by a relatively benign treatment. An example might be cancer surgery where the event is cancer spread to adjacent lymph nodes. A positive test for lymph node involvement would lead to a lymph node dissection, which is a relatively benign procedure; a false negative would lead to a cancer recurrence. An aggressive infection that is easily cured with antibiotics if found early would be a second example. Two tests are available: one with a specificity of 95% and a sensitivity of 50% and the other has a sensitivity of 95% and specificity of 50%. As the harms of missing disease far outweigh the harms of unnecessary treatment, we would favor the test with a higher sensitivity. However, the Brier score favors the more specific test (Table 1), because a majority of patients do not have the disease. The rank ordering of tests is unaffected by the method of calculating the Brier score, although method 1—using 0 and 1 as the predicted probability from the test—gives more extreme results than method 2, which assumes that the output of a binary test is negative and positive predictive values. Conversely, the net benefit for low threshold probabilities—appropriate given the clinical context where sensitivity is favored—is greater for the highly sensitive test (0.1689 versus 0.0979 at a threshold of 5%; Table 1).

**Table 1:** Performance characteristics of binary tests and continuous prediction models with various degrees of miscalibration. All values given were calculated directly from the formulae in the text and independently verified using a simulation approach (Appendix)

					Net benefit		
Test	Specificity	Sensitivity	AUC	Brier score	Threshold: 5%	Threshold: 10%	Threshold: 20%
Binary tests							
Assume all negative	100%	0%	0.500	0.2000	0.0000	0.0000	0.0000
Assume all positive	0%	100%	0.500	0.8000	0.1579	0.1111	0.0000
Highly specific	95%	50%	0.725	0.1400* 0.1169^†^	0.0979	0.0956	0.0900
Highly sensitive	50%	95%	0.725	0.4100* 0.1386^†^	0.1689	0.1456	0.0900
Continuous prediction models							
Well calibrated	–	–	0.75	0.1386	0.1595	0.1236	0.0716
Overestimating risk	–	–	0.75	0.1708	0.1583	0.1160	0.0423
Underestimating risk	–	–	0.75	0.1540	0.1483	0.0986	0.0413
Severely underestimating risk	–	–	0.75	0.1760	0.0921	0.0372	0.0076

AUC, Brier score, and net benefit for various threshold probabilities corresponding to binary tests and continuous prediction models with various degrees of miscalibration predicting an outcome with prevalence of 20%, as shown in Fig. 1. Higher values of AUC and net benefit are desirable whereas lower values of the Brier score are desirable

*Method 1 calculation: binary test is considered to produce probabilities of 1 and 0 for a positive and negative test, respectively

^†^Method 2 calculation: binary test is considered to produce probabilities of the positive predictive value and 1 − negative predictive value for a positive and negative test, respectively

These findings hold if we reverse the situation, with disease prevalence above 50%, and a clinical context where specificity is favored, for instance, if unnecessary treatment is very harmful. For instance, if prevalence is 70%, the Brier score favors a binary test with sensitivity of 90% and specificity of 80% over the test with 80% sensitivity and 90% specificity (0.1090 versus 0.1207, method 2). Net benefit again selects the preferable test. The net benefit at a threshold probability of 80% is 0.3900 and 0.4400 for the more sensitive and more specific test, respectively.

#### Comparison to default strategies of assuming all or no patients are positive

The alternative to using a prediction model or binary test is to use a default strategy of considering all patients are either positive or negative (i.e., assigning all patients a predicted risk of 1 or 0, respectively). In our cancer surgery example, where it is important to identify the low prevalence event of lymph node involvement, assuming that all patients are positive (e.g., a more extended surgical resection in all cases) might be a strategy worth considering, whereas assuming all patients are negative (e.g., no extended resections) is harmful. As shown in Table 1, the Brier score for the former is extremely poor whereas the Brier score for the latter is favorable. On the other hand, assuming that all patients are positive appropriately has superior net benefit to assuming all patients are negative, which by definition has a net benefit of 0 at all thresholds.

#### Binary tests versus prediction models

Consider the primary example where prevalence is 20%, and missed treatment is much more harmful than unnecessary treatment of a false positive. Let us assume that it has been determined that, accordingly, sensitivity needs to be above 90%. Take two binary tests, one with a sensitivity of 50% and specificity of 95% and the other with a sensitivity in the desirable range (95%) and a specificity of 50%, thus AUC of 0.725 for both. Compare these with a prediction model that has an AUC of 0.75 which overestimates risk. Note in particular that for a prediction model with an AUC of 0.75 where predictions are normally distributed on the logit scale, a cut point with a sensitivity of 95% will be associated with a specificity of close to 25%, far inferior to the more sensitive binary test. The Brier score incorrectly favors the highly specific test. Net benefit provides the correct ordering of tests by ranking the highly sensitive binary test the highest, followed by the continuous model and with the highly specific binary test coming in last (5% threshold net benefit is 0.1689, 0.1583, and 0.0979, respectively). Indeed, the specific test has lower net benefit than the strategy of assuming all patients are positive, a strategy that is clinically sensible but is strongly disfavored by Brier score (Table 1).

#### Brier score and miscalibration

The Brier score inherently incorporates calibration, such that for two models miscalibrated in the same direction, the score is positively associated with the degree of miscalibration. However, the Brier score can rank models in an undesirable way where miscalibration is in opposite directions than what is clinically favorable.

Table 1 shows that when prevalence is 20%, the well-calibrated model had the best Brier score and net benefit (0.1386 and 0.1595, respectively). However, the model that overestimates risk has a poorer Brier score (0.1708) than the model that underestimates risk (0.1540). This is problematic because we have a clinical scenario where sensitivity is key, and hence, we would rather overestimate risk. For net benefit, low threshold probabilities are of interest for the clinical scenario where sensitivity is critical. At these thresholds, net benefit favors the model that overestimates risk compared to models that underestimate risk (Table 1).

It is easily shown that these findings are consistent if specificity is favored and prevalence is above 50%: Brier score but not net benefit favors the model that is miscalibrated in the less desirable direction.

## Discussion

We found that although Brier score does have some desirable properties—for instance, as it is proper scoring rule, a well-calibrated model has a better Brier score than a miscalibrated model with similar discrimination—the Brier score does not perform well in several common situations. We have demonstrated that the Brier score will favor a test with high specificity where the clinical context requires high sensitivity if it is the case that prevalence is low. We also found that continuous models have better Brier scores than binary tests even if the test clearly has superior properties and the model badly miscalibrated. The inappropriate rank ordering of binary tests extended to default strategies of assuming that all or no patient is positive, so that the clinically sensible approach of treating all patients at risk of an easily curable but potentially fatal disease had a worse Brier score than the use of a badly miscalibrated prediction model that severely underestimates risk. We have also demonstrated that the Brier score may inappropriately select between models miscalibrated in opposite directions.

In contrast, net benefit always gave a rank ordering that was consistent with any reasonable evaluation of the preferable test or model in a given clinical situation. For instance, a sensitive test had a higher net benefit than a specific test where sensitivity was clinically important. It is perhaps not surprising that a decision-analytic technique gives results that are in accord with clinical judgment because clinical judgment is “hardwired” into the decision-analytic statistic.

We might also note that Table 1 clearly supports the current consensus that discrimination is an inadequate statistic: the AUC of the two binary tests were identical as were those of the four prediction models, even though there is an unambiguous preference for one or other tests or models in all of the clinical scenarios.

The consequence of demonstrating that the Brier score gives an inappropriate rank ordering of tests and models in several scenarios—scenarios that could hardly be described as extreme or unusual—is that the Brier score cannot be considered to have general applicability for evaluating clinical utility. There might be a case for defining where Brier score is and is not appropriate for assessing clinical utility, if it were not for the fact that an alternative, net benefit, is simple to calculate, also fulfills the criterion for being a proper scoring rule, and chooses the favored test or model in all of our example scenarios. It is true that the Brier score gives a single statistic, whereas net benefit is calculated across a range, but this only complicates analysis if the rank order of tests and models varies depending on threshold probability, within a reasonable range. It is also true that net benefit does require additional external information—the appropriate range of threshold probabilities—information that is subjective. But the degree of external information required is minor and the subjectivity required is an inherent part of clinical decision-making. For instance, an opinion that, say, an untreated infection is 19 times worse than unnecessary antibiotics is essential for clinical implementation of a prediction model that gives the risk of infection (i.e., give antibiotics if risk is ≥ 5%).

A possible counterargument to our paper is that the Brier score is an overall measure of the quality of predictions that is independent of any given clinical scenario. We agree with this point and would like to stress that we are not making a general argument against the Brier score. Indeed, we ourselves have used Brier scores, for instance, when comparing different variable parameterizations in the early stages of model development. Our aim here focuses on clinical practice: we show that the Brier score should not be used to evaluate whether a test or model should be used clinically, or to determine which of two competing clinical models or tests is preferable.

Another limitation to our approach is that we did not investigate alternative decision-analytic variants of the Brier score which have been proposed [19]. These alternative variants allow for differential penalization for false-positive and false-negative misclassifications. However, these variants do not appear to have ever been used in practice and methodological references are rare. It may well be that a decision-analytic Brier score has good properties, but this does not affect our main point. The Brier score referenced in the literature to determine the clinical utility of a test, used in practice (for example, on the basis of a Brier score, a model was recommended to “assist clinicians in individual dosage of their patients” [9]), described in textbooks [8], and coded into software has undesirable properties in several common scenarios.

We have proposed net benefit as a suitable performance assessment alternative. That said, this measure is not without its own limitations, in particular, the assumption that the benefit and harms of treatment do not vary importantly between patients independently of preference.

## Conclusion

The Brier score does not appropriately evaluate the clinical utility of diagnostic tests or prediction models. We advocate the use of decision-analytic measures such as net benefit.

## Appendix

### Simulation verification

Through simulation, we have generated each of the scenarios displayed in Table 1. We generated a data set with an arbitrarily large sample size (*n* = 1,000,000) and binary disease status with the desired prevalence, generated binary test status based on prespecified sensitivity and specificities with the outcomes, and generated continuous prediction models. The prediction models generated using the simulated data were compared on the basis of their discrimination and calibration. We then calculated AUC, Brier score, and net benefit based on the simulated disease status, binary test status, and continuous prediction models.

### Binary tests

We randomly generated disease status using a binomial distribution with the desired event rate. We then randomly generated the test status among those with the disease using a binomial distribution with the probability of a positive test equaling the sensitivity. Among those without the disease, we randomly generated the test status using a binomial distribution with the probability of a positive test equaling 1-specificity. The method 1 Brier score, AUC, and net benefit were calculated using the binary test status and disease status. When generating the Brier score under method 2, we added the following step: generating the probability of disease by test status (PPV and NPV). We calculated the probability of disease among those with a positive test and negative test, separately, and calculated the Brier score using the updated probability of disease in place of the binary test outcome.

### Continuous prediction models

Four prediction models were created: a well-calibrated model and, alternatively, models that overestimated or underestimated risk. We generated an arbitrarily large dataset with the desired event rate of 20% and a difference of a 1 standard deviation increase among those with the disease. The model was then miscalibrated to various degrees by shifts on the intercept coefficient. The AUC, Brier score, and net benefit were calculated using the continuous predicted value and disease status.

## Abbreviations

AUC: Area under the curve
NRI: Net reclassification improvement
